# Chromosome studies in the aquatic monocots of Myanmar: A brief review with additional records

**DOI:** 10.3897/BDJ.2.e1069

**Published:** 2014-05-13

**Authors:** Yu Ito, Nobuyuki Tanaka

**Affiliations:** † University of Canterbury, Christchurch, New Zealand; ‡ Kochi Prefectural Makino Botanical Garden, Kochi, Japan

**Keywords:** Aquatic plants, chromosome counts, *
Limnocharis
*, Myanmar, *
Potamogeton
*, *
Sagittaria
*

## Abstract

Myanmar (Burma) constitutes a significant component of the Indo-Myanmar biodiversity hotspot, with elements of the Indian, the Indochina, and the Sino-Japanese floristic regions, yet thus far only a few reliable sources of the country's flora have been available. As a part of a contribution for the floristic inventory of Myanmar, since it is important in a floristic survey to obtain as much information as possible, in addition to previous two reports, here we present three more chromosome counts in the aquatic monocots of Myanmar: *Limnocharis flava* with 2n = 20, *Sagittaria trifolia* with 2n = 22 (Alismataceae), and *Potamogeton distinctus* × *Potamogeton nodosus* with 2n = 52 (Potamogetonaceae); the third one is new to science. A brief review of cytological researches in the floristic regions' 45 non-hybrid aquatic monocots plus well investigated two inter-specific hybrids that are recorded in Myanmar is given, indicating that the further works with a focus on species in Myanmar that has infra-specific chromosome variation in the floristic regions will address the precise evolutionary history of the aquatic flora of Myanmar.

## Introduction

With its wealth of plant diversity, Myanmar (Burma) constitutes a significant component of the Indo-Myanmar biodiversity hotspot with elements of the India, the Indochina, and the Sino-Japanese floristic regions (ca, 13,500 vascular plants: Van Dijk et al. 2004; Tanaka 2010). Yet, while neighboring countries’ floristic diversity has been exposed through international projects, such as Flora of China, Flore du Cambodge, du Laos et du Vietnam, and Flora of Thailand, thus far no reliable sources of Myanmar’s flora have been published except a checklist of spermatophytes contributed by Kress et al. (2003). In order to revise the flora of Myanmar, a decade-long continuous inventory has been conducted by Japanese botanists (Tanaka 2005), which thus far partly contributed a local checklist (Mt. Popa: Tanaka et al. 2006) and a taxon-specific checklist (aquatic plants: Ito and Barfod 2014).

The aim of floristic research is not only to count the total number of species but also to evaluate the native flora’s evolutionary origins by comparing with related floristic regions. From this aspect, it is useful to obtain as much information as possible, e.g., chromosome data (Sanders et al. 1983). This is especially important for floristic surveys for aquatic plants, in which infra- or inter-specific chromosome variation is widely known (Les and Philbrick 1993). The proportion of species for which the chromosome number is known is less than 1% in some little-collected tropical areas (Stace 2000), probably including the southeast Asian country of Myanmar.

Aquatic plants, which is polyphyletically evolved in fern and fern allies, basal angiosperms, monocots, and eudicots, is known as having numerous chromosomal variation, thus an excellent model for this aim. Here, in addition to the previous contributions of chromosome counts for new or noteworthy aquatic plants from Myanmar (*Najas tenuis*: Ito et al. 2014b; *Nechamandra alternifolia*: Ito et al. 2009), we present three more chromosome counts for the aquatic monocots of Myanmar: *Limnocharis flava* (Alismataceae), *Sagittaria trifolia* (Alismataceae), and *Potamogeton distinctus* × *Potamogeton nodosus* (Potamogetonaceae). A brief review of cytological researches in 45 non-hybrid aquatic monocots plus two well-investigated inter-specific *Potamogeton* hybrids in Myanmar is also given with a broad focus on those distributed in neighboring areas, i.e., the Indian, the Indochina, and the Sino-Japanese floristic regions.

## Materials and methods

### Chromosome observation

Plant materials of *Limnocharis flava* (Alismataceae), *Sagittaria trifolia* (Alismataceae), *Najas tenuis* (Hydrocharitaceae), *Nechamandra alternifolia* (Hydrocharitaceae), and *Potamogeton distinctus* × *Potamogeton nodosus* (Potamogetonaceae) were collected in the expeditions to Myanmar (Bago Division and Shan State) in 2008. The collections were rigorously identified based on morphological characters using the original protologues as well as a previous taxonomic treatment by Cook (1996). *Potamogeton distinctus* × *Potamogeton nodosus* (Potamogetonaceae) was identified by DNA barcoding method (Ito et al. 2014a). The first set of the voucher specimens was retained in Forest Department Office, Ministry of Environmental Conservation and Forestry, Union of Myanmar (RAF); the duplicates are deposited in two Japanese herbaria: Makino Botanical Garden (MBK) and the University of Tokyo (TI).

Root tips collected in the field were pretreated with 0.002 M 8-hydroxyquinoline at 4 °C in 12 h, and fixed with freshly mixed Carnoy’s fixative (3: 1 ethyl alcohol: acetic acid) for at least 30 min, and then preserved at 4 °C in 12 h. For microscopic observation, root tips were soaked in 1 N HCl for 1 h followed by 10 min at 60 °C. After being immersed in tap water, the materials were stained in a drop of 1.5% orcein acetate solution on a slide glass in 5 min., and then squashed. Then somatic chromosome numbers of the three taxa were obtained by light microscopic examination. For each species, at least two cells were used to confirm the numbers.

Distribution for each species follows Ito and Barfod (2014).

### Literature review

Chromosome researches for aquatic monocots of Myanmar were reviewed with a broad focus on Myanmar and related floristic regions, i.e., the Indian, the Indochina, and the Sino-Japanes floristic regions. The focal species include 45 non-hybrid aquatic monocots listed in Ito and Barfod (2014), Ito et al. (2014a) as well as well-investigated two inter-specific *Potamogeton* hybrids (Ito et al. 2014a). Initial literature search was carried out with Fedorov (1969) as well as Index to Plant Chromosome Numbers (Missouri Botanical Garden, http://mobot.mobot.org/W3T/Search/ipcn.html), followed by extensive literature review with original references. For some species, mostly cosmopolitan ones, only a few representative literature references are given for each chromosome number. Since a comprehensive cytological review was given for aquatic plants (Les and Philbrick 1993), including almost all the taxa listed in the present study, our literature review focused on literature published in 1993 or later. Due to incapability of original references, some rare chromosome counts are not included; those references are mostly published in 1970 or earlier, and written not in English. No detailed references are given for Potamogetonaceae and *Ruppia* because an exhaustive cytological review was published by Kaplan et al. (2013), Talavera et al. (1993).

## Checklists

### Chromosome counts for the aquatic monocots of Myanmar

#### Alismatales

#### Alismataceae

#### Limnocharis

Bonpl., 1808

#### Limnocharis flava

(L.) Buchenau, 1868

##### Materials

- **Type status:** Other material. **Occurrence:** recordedBy: Y. Ito; **Location:** country: Myanmar; stateProvince: Bago; municipality: Pyat Township; locality: along the roadside, paddy field, ca. 30 km east of Pyat; verbatimLatitude: 18°49'44"N; verbatimLongitude: 95°18'06"E; **Event:** eventDate: 7 Dec 2008; **Record Level:** collectionID: N. Tanaka & al. 080776; institutionCode: MBK, RAF, TI

##### Distribution

Native to Americas; naturalized to tropical Asia.

##### Notes

**Figure 1.**
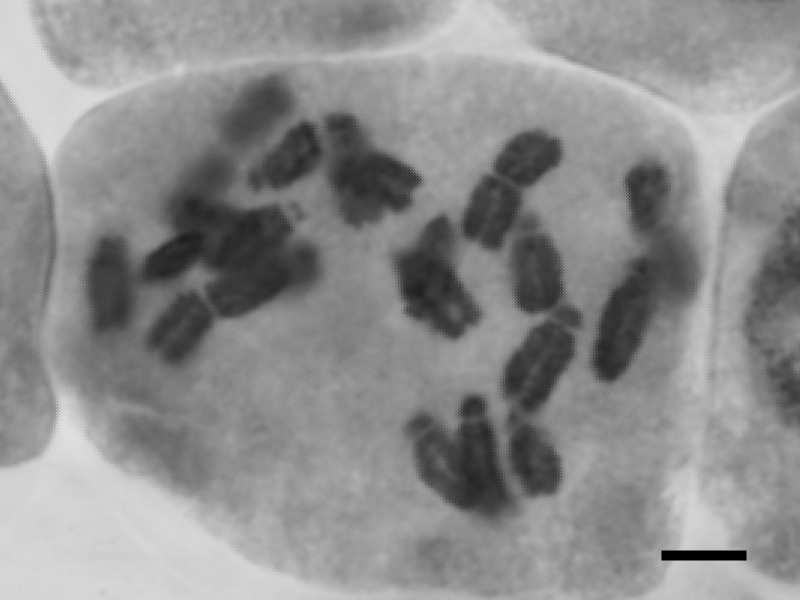
Somatic chromosome of *Limnocharis flava*. Bar indicates 5 μm.

Chromosome counts: 2n = 20 (Fig. 1; obtained in this study).

#### Sagittaria

L., 1753

#### Sagittaria trifolia

L., 1753

##### Materials

- **Type status:** Other material. **Occurrence:** recordedBy: Y. Ito; **Location:** country: Myanmar; stateProvince: Shan; verbatimLocality: Pindaya; verbatimLatitude: 20°59'57"N; verbatimLongitude: 96°39'59"E; **Event:** eventDate: 1 Dec 2008; **Record Level:** collectionID: N. Tanaka & al. 080623

##### Distribution

Bangladesh, Bhutan, China (nationwide), India (nationwide), Indonesia (Borneo, Java, Sulawesi), Japan, Malaysia (Peninsular), Myanmar, Nepal, Pakistan, Philippines, Thailand; Oceania.

##### Notes

**Figure 2.**
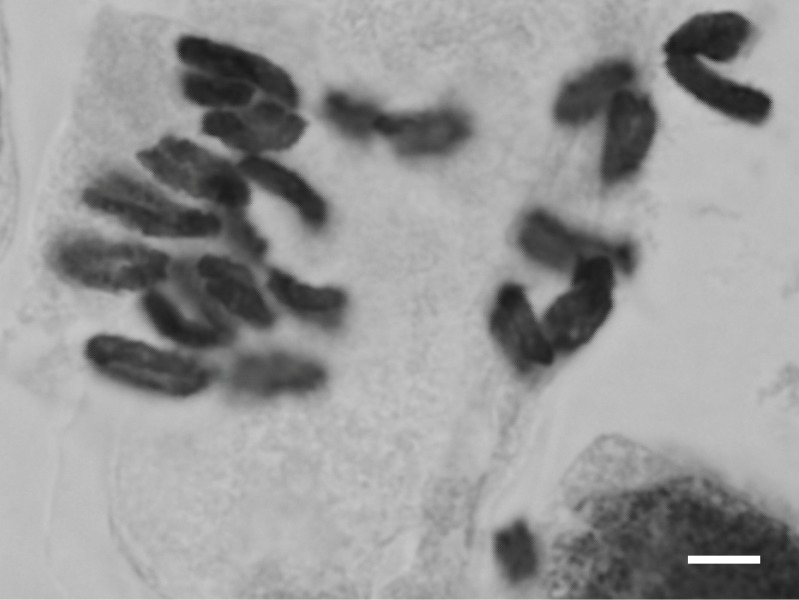
Somatic chromosome of *Sagittaria trifolia*. Bar indicates 5 μm.

Chromosome counts: 2n = 22 (Fig. 2; obtained in this study).

#### Hydrocharitaceae

#### Najas

L., 1753

#### Najas tenuis

Magnus, 1870

##### Materials

- **Type status:** Other material. **Occurrence:** recordedBy: Y. Ito; **Location:** country: Myanmar; stateProvince: Shan; verbatimLocality: Inlay Lake, Nyaung Shwe Township; verbatimLatitude: 20°32'02"N; verbatimLongitude: 96°53'53"E; **Event:** eventDate: 3 Dec 2008; **Record Level:** collectionID: N. Tanaka & al. 080642; institutionCode: MBK, RAF, TI

##### Distribution

India (Central, Southern), Myanmar, Sri Lanka.

##### Notes

**Figure 3.**
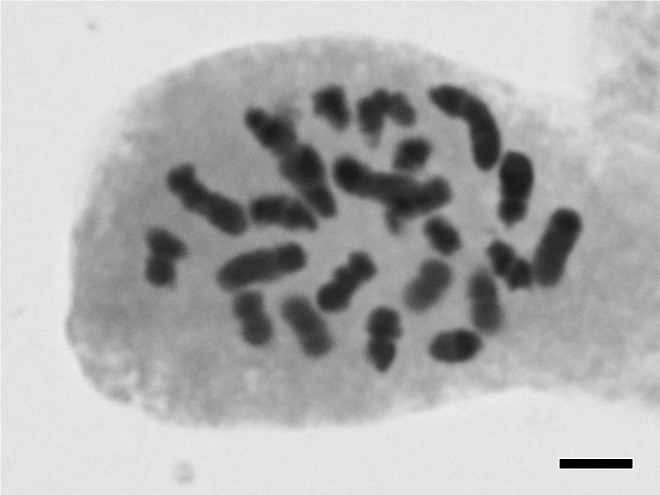
Somatic chromosome of *Najas tenuis*. Bar indicates 5 μm.

Chromosome counts: 2n = 24 (Fig. 3; After Ito et al. 2014b; reproduced with publisher's permission).

#### Nechamandra

Planch., 1849

#### Nechamandra alternifolia

(Roxb.) Thwaites, 1864

##### Materials

- **Type status:** Other material. **Occurrence:** recordedBy: Y. Ito; **Location:** country: Myanmar; stateProvince: Shan; verbatimLocality: Near Yae Aye Kan Dam, Yae Aye Kan, Kalaw Township; verbatimLatitude: 20°35'37"N; verbatimLongitude: 96°31'46"E; **Event:** eventDate: 26 Nov 2008; **Record Level:** collectionID: N. Tanaka & al. 080058; institutionCode: MBK, RAF, TI

##### Distribution

Bangladesh, China (Southern), India (Eastern, Northern, Southern), Myanmar, Nepal, Sri Lanka, Thailand, Vietnam; Yemen, and Sudan.

##### Notes

**Figure 4.**
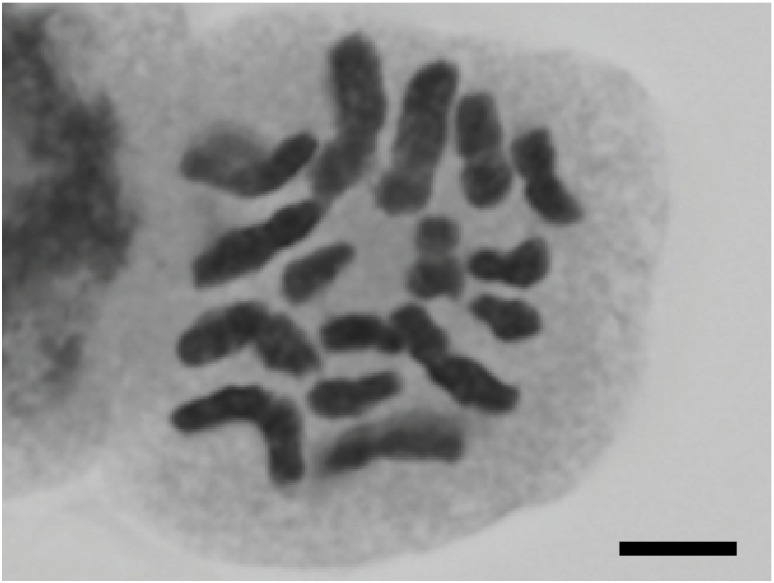
Somatic chromosome of *Nechamandra alternifolia*. Bar indicates 5 μm.

Chromosome counts: 2n = 16 (Fig. 4; After Ito et al. 2009; reproduced with publisher's permission).

#### Potamogetonaceae

#### Potamogeton

L., 1753

#### Potamogeton distinctus A. Benn. × P. nodosus Poir.

##### Materials

- **Type status:** Other material. **Occurrence:** recordedBy: Y. Ito; **Location:** country: Myanmar; stateProvince: Shan; verbatimLocality: Inle Lake; verbatimLatitude: 20°27'28"N; verbatimLongitude: 96°50'37"E; **Event:** eventDate: 4 Dec 2008; **Record Level:** collectionID: N. Tanaka & al. 080662; institutionCode: MBK, RAF, TI

##### Notes

**Figure 5.**
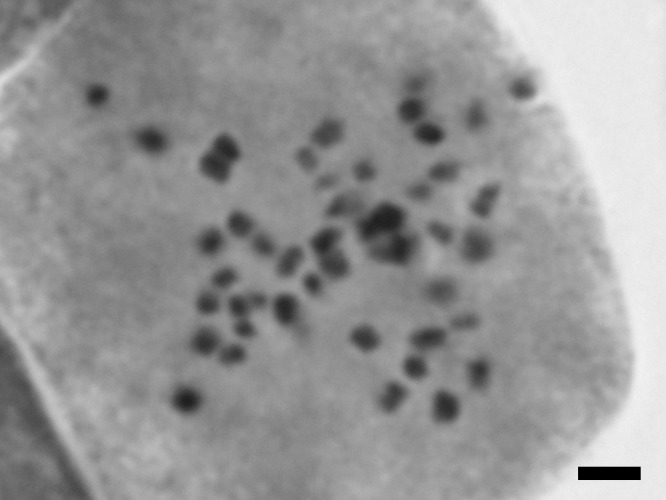
Somatic chromosome of *Potamogeton distinctus* × *Potamogeton nodosus*. Bar indicates 2.5 μm.

Chromosome counts: 2n = 52 (Fig. 5; obtained in this study). The chromosome count for this taxon is new to science.

## Analysis

The chromosome counts given for 45 non-hybrid species of aquatic monocots of Myanmar as well as well-investigated two *Potamogeton* hybrids among them were reviewed with a focus on infra-specific chromosome variation (Table 1). The cited literature references also include chromosome counts obtained from related floristic regions, i.e., the Indian, the Indochina, and the Sino-Japanese floristic regions. For widespread species, cytological information from other regions is cited.

## Discussion

Of 45 non-hybrid aquatic monocots and two interspecific hybrids among them, more than two thirds have no chromosome variation. Meanwhile, the following nine species have infra-specific chromosome variation, i.e., *Acorus calamus*, *Cryptocoryne crispatula*, *Blyxa echinosperma*, *Hydrilla verticillata*, *Najas graminea*, *Ottelia alismoides*, *Vallisneria spiralis*, *Monochoria hastata*, and *Monochoria vaginalis* (Table 1). Among the cytologically variable aquatic monocots are *Acorus calamus*, *Ottelia alismoides*, *Vallisneria spiralis*, and *Monochoria vaginalis*, for which unique chromosome counts are obtained from each floristic region. Myanmar is known as including borders among the Indian, the Indochina, and the Sino-Japanese floristic regions (Tanaka 2010), yet in the aquatic flora, it is unknown which flora is more influenced. Future research with a focus on such species will address this issue.

*Potamogeton* is known as having numerous inter-specific hybrids, and each parental combination is varied from intra-ploidy crosses to inter-ploidy ones (Kaplan et al. 2013). The present study revealed *Potamogeton distinctus* × *Potamogeton nodosus* as another intra-ploidy hybrid of *Potamogeton* at tetraploid level.

## Supplementary Material

XML Treatment for Alismatales

XML Treatment for Alismataceae

XML Treatment for Limnocharis

XML Treatment for Limnocharis flava

XML Treatment for Sagittaria

XML Treatment for Sagittaria trifolia

XML Treatment for Hydrocharitaceae

XML Treatment for Najas

XML Treatment for Najas tenuis

XML Treatment for Nechamandra

XML Treatment for Nechamandra alternifolia

XML Treatment for Potamogetonaceae

XML Treatment for Potamogeton

XML Treatment for Potamogeton distinctus A. Benn. × P. nodosus Poir.

## Figures and Tables

**Table 1.** The chromosome counts given for 45 non-hybrid species of aquatic monocots of Myanmar as well as well-investigated two *Potamogeton* hybrids among them. Those recorded from neighboring regions are also provided. The species that have no chromosome counts anywhere in the world are shown with n/a. For some species, mostly cosmopolitan ones, only a few representative literature references are given for each chromosome number. Note that due to incapability of original references, some rare chromosome counts are not included in this table: 2n = 18, 42, 48 for *Acorus calamus*; 2n = 18, 22 for *Acorus gramineus* (Acoraceae), 2n = 28 for Pistia stratiotes var. cuneata Engl.; 2n = 28 for Pistia stratiotes var. spathulata (Michx.) Engl.; 2n = 20, 50, 60, 80 for *Lemna aequinoctialis*; 2n = 44 for *Lemna trisulca*; 2n = 30, 50 for *Spirodela polyrrhiza* (Araceae); n = 14 (2n = 28), 2n = 10, 12 for *Alisma plantago-aquatica*; 2n = 22 for *Caldesia parnassifolia*; 2n = 26, 39 for *Limnocharis flava*; 2n = 22 for Sagittaria trifolia var. longiloba (Turr.) Mak.; 2n = 22 for Sagittaria trifolia var. sinensis Sims; 2n = 22 for Sagittaria trifolia var. edulis (Sieb.) Ohwi (Alismataceae); 2n = 24 for *Blyxa aubertii*; 2n = 60 for *Najas marina*; 2n = 12+1B for Najas marina var. intermedia (Gorski) A. Braun; 2n = 22, 52, 72, 88, 132 for *Ottelia alismoides*; 2n = 16, 22, 28, 33 for *Vallisneria spiralis* (Hydrocharitaceae); 2n = 64 for *Eichhornia crassipes*; 2n = 26, n = 40 (2n = 80) for *Monochoria vaginalis* (Pontederiaceae); 2n = 60 for *Typha angustifolia* (Typhaceae). Also refer to previous cytological reviews (aquatic plants: Les and Philbrick 1993; Potamogetonaceae: Kaplan et al. 2013; *Ruppia*: Talavera et al. 1993).

Order	Family	Species	Chromo-some number	Floristic region				
				Indian	Myanmar	Indo-china	Sino-Japanese	Others
Acorales	Acoraceae	*Acorus calamus* L.	2n = 24	Subramanian and Munian (1988)				Chepinoga et al. (2008)
Acorales	Acoraceae	*Acorus calamus* L.	2n = 35					Krahulcová (2003)
Acorales	Acoraceae	*Acorus calamus* L.	2n = 36					Packer and Ringius (1984)
Acorales	Acoraceae	*Acorus calamus* L.	2n = 44				Wang et al. (2001)	
Acorales	Acoraceae	*Acorus calamus* L.	2n = 45	Ramachandran (1978)				
Acorales	Acoraceae	*Acorus calamus* L.	2n = 66				Wang et al. (2001)	
Acorales	Acoraceae	*Acorus gramineus* Sol. ex Aiton	2n = 24				Wang et al. (2001)	
Acorales	Araceae	*Cryptocoryne crispatula* Engl.	2n = 36	Arends et al. (1982)				
Acorales	Araceae	*Cryptocoryne crispatula* Engl.	2n = 54	Jacobsen (1977)				
Acorales	Araceae	*Cryptocoryne cruddasiana* Prain	n/a					
Acorales	Araceae	*Pistia stratiotes* L.	2n = 28	Ramachandran (1978), Subramanian and Munian (1988)				
Acorales	Araceae	*Landoltia punctata* (G. Mey.) Les & D.J. Crawford	n/a					
Acorales	Araceae	*Lemna aequinoctialis* Welw.	2n = 40	Urbanska-Worytkiewicz (1975) (*Lemna perpusilla* Torr.)			Beppu et al. (1985)	
Acorales	Araceae	*Lemna trisulca* L.	2n = 20					Urbanska-Worytkiewicz (1975)
Acorales	Araceae	*Lemna trisulca* L.	2n = 40					Urbanska-Worytkiewicz (1975)
Acorales	Araceae	*Lemna trisulca* L.	2n = 60					Urbanska-Worytkiewicz (1975); Löve and Löve (1981)
Acorales	Araceae	*Lemna trisulca* L.	2n = 80					Urbanska-Worytkiewicz (1975)
Acorales	Araceae	*Spirodela polyrrhiza* (L.) Schleid.	2n = 40					Löve and Löve (1981), Al-Bermani et al. (1993)
Acorales	Araceae	*Spirodela polyrrhiza* (L.) Schleid.	2n = 42					Chepinoga et al. (2008)
Acorales	Araceae	*Spirodela polyrrhiza* (L.) Schleid.	2n = 80					Geber and Schweizer (1988)
Acorales	Araceae	*Wolffia globosa* (Roxb.) Hartog &Plas	n/a					
Alismatales	Alismataceae	*Alisma plantago-aquatica* L.	2n = 14	Mehra and Pandita (1984)			Wang et al. (1987); Uchiyama (1989) (var. *Alismaplantago-aquaticaorientale* Samuel)	
Alismatales	Alismataceae	*Caldesia parnassifolia* (Bassi ex L.) Parl.	n/a					
Alismatales	Alismataceae	*Limnocharis flava* (L.) Buchenau	2n = 20		This study		Uchiyama (1989)	Davidse (1981), Forni-Martins and Calligaris (2002)
Alismatales	Alismataceae	*Sagittaria trifolia* L.	2n = 22		This study		Uchiyama (1989); (var. *Sagittariatrifoliaedulis* (Sieb.) Ohwi)	
Alismatales	Hydrocharita	*Blyxa aubertii* Rich.	2n = 40				Uchiyama (1989)	
Alismatales	Hydrocharita	*Blyxa echinosperma* (C.B. Clarke) Hook. f.	2n = 42				Wang (1986)	
Alismatales	Hydrocharita	*Blyxa echinosperma* (C.B. Clarke) Hook. f.	2n = 74				Uchiyama (1989)	
Alismatales	Hydrocharita	*Blyxa japonica* (Miq.) Maxim. ex Asch. & Gürke	2n = 42				Harada (1956)	
Alismatales	Hydrocharita	*Blyxa japonica* (Miq.) Maxim. ex Asch. & Gürke	2n = 72				Uchiyama (1989)	
Alismatales	Hydrocharita	*Egeria densa* (Planch.) Casp.	2n = 46				Uchiyama (1989), Nakata and Nagai (1998)	
Alismatales	Hydrocharita	*Egeria densa* (Planch.) Casp.	2n = 48					Löve and Löve (1961)
Alismatales	Hydrocharita	*Elodea nuttallii* (Planch.) H. St. John	2n = 48					Simpson (1986)
Alismatales	Hydrocharita	*Hydrilla verticillata* (L. f.) Royle	2n = 16	Chaudhuri and Sharma (1978), Pandita and Mehra (1984)			Wang (1986), Uchiyama (1989), Langeland et al. (1992)	Langeland et al. (1992)
Alismatales	Hydrocharita	*Hydrilla verticillata* (L. f.) Royle	2n = 24	Chaudhuri and Sharma (1978)			Langeland et al. (1992), Nakata and Nagai (1998)	Langeland et al. (1992)
Alismatales	Hydrocharita	*Hydrilla verticillata* (L. f.) Royle	2n = 32				Langeland et al. (1992)	Langeland et al. (1992)
Alismatales	Hydrocharita	*Hydrocharis dubia* (Blume) Backer	2n = 16	Pandita and Mehra (1984)			Uchiyama (1989)	
Alismatales	Hydrocharita	*Najas graminea* Delile	2n = 12				You et al. (1991)	
Alismatales	Hydrocharita	*Najas graminea* Delile	2n = 24				Wang (1985), Uchiyama (1989)	
Alismatales	Hydrocharita	*Najas graminea* Delile	2n = 36				Uchiyama (1989)	
Alismatales	Hydrocharita	*Najas indica* (Willd.) Cham.	n/a					
Alismatales	Hydrocharita	*Najas marina* L.	2n = 12				Wang (1985), Uchiyama (1989)	
Alismatales	Hydrocharita	*Najas marina* L.	2n = 24					Viinikka et al. (2008)
Alismatales	Hydrocharita	*Najas tenuis* Magnus	2n = 24		Ito et al. (2014b)			
Alismatales	Hydrocharita	*Nechamandra alternifolia* (Roxb.) Thwaites	2n = 16	Sharma and Chatterjee (1967)	Ito et al. (2009)			
Alismatales	Hydrocharita	*Ottelia alismoides* (L.) Pers.	2n = 44				Harada (1956), Uchiyama (1989)	
Alismatales	Hydrocharita	*Ottelia alismoides* (L.) Pers.	2n = 66	Chaudhuri and Sharma (1978)				
Alismatales	Hydrocharita	*Ottelia alismoides* (L.) Pers.	2n = 68	Chaudhuri and Sharma (1978)				
Alismatales	Hydrocharita	*Ottelia cordata* (Wall.) Dandy	n/a					
Alismatales	Hydrocharita	*Vallisneria spiralis* L.	2n = 20				Wang (1986)	
Alismatales	Hydrocharita	*Vallisneria spiralis* L.	2n = 24	Chaudhuri and Sharma (1978)				
Alismatales	Hydrocharita	*Vallisneria spiralis* L.	2n = 30	Chaudhuri and Sharma (1978)				
Alismatales	Hydrocharita	*Vallisneria spiralis* L.	2n = 40	Chaudhuri and Sharma (1978), Sarkar et al. (1980)				
Alismatales	Aponogetona	*Aponogeton lakhonensis* A. Camus	n/a					
Alismatales	Potamogetona	*Potamogeton crispus* L.	2n = 52				Kaplan et al. (2013)	Kaplan et al. (2013)
Alismatales	Potamogetona	*Potamogeton crispus* L.	2n = 56				Nakata and Nagai (1998)	
Alismatales	Potamogetona	*Potamogeton distinctus* A. Benn.	2n = 52				Kaplan et al. (2013)	
Alismatales	Potamogetona	*Potamogeton distinctus* A. Benn. × *Potamogeton nodosus* Poir.	2n = 52		This study			
Alismatales	Potamogetona	*Potamogeton maackianus* A Benn.	2n = 52				Kaplan et al. (2013)	
Alismatales	Potamogetona	*Potamogeton maackianus* A Benn.	2n = 56			Kaplan et al. (2013)	Uchiyama (1989), Kaplan et al. (2013)	
Alismatales	Potamogetona	Potamogeton × malainoides Miki	2n = 52				Kaplan et al. (2013)	
Alismatales	Potamogetona	*Potamogeton lucens* L.	2n = 52				Kaplan et al. (2013)	Kaplan et al. (2013)
Alismatales	Potamogetona	*Potamogeton nodosus* Poir.	2n = 52					Kaplan et al. (2013)
Alismatales	Potamogetona	*Potamogeton octandrus* Poir.	2n = 28				Uchiyama (1989), Nakata and Nagai (1998), Kaplan et al. (2013)	
Alismatales	Potamogetona	*Potamogeton wrightii* Morong	2n = 52				Kaplan et al. (2013)	
Alismatales	Potamogetona	*Stuckenia pectinata* (L.) Börner	2n = 78	Kaplan et al. (2013)				Kaplan et al. (2013)
Alismatales	Potamogetona	*Stuckenia pectinata* (L.) Börner	2n = 84				Uchiyama (1989)	
Alismatales	Ruppiaceae	*Ruppia maritima* L.	2n = 20	Ito et al. (2010)		Ito et al. (2010)	Ito et al. (2010)	Van Vierssen et al. (1981)
		*Ruppia maritima* L.	2n = 40				Harada (1956), Ito et al. (2010)	Ito et al. (2010)
Asparagales	Amaryllidaceae	*Crinum thaianum* J. Schul.	n/a					
Commelinales	Pontederiaceae	*Eichhornia crassipes* (Mart.) Solms	2n = 32					Pedrosa et al. (1999)
Commelinales	Pontederiaceae	*Monochoria hastata* (L.) Solms	2n = 28	Patwary et al. (1989)				
Commelinales	Pontederiaceae	*Monochoria hastata* (L.) Solms	2n = 80	Patwary et al. (1989)				
Commelinales	Pontederiaceae	*Monochoria vaginalis* (Burm.f.) C. Presl ex Kunth	2n = 24	Christopher (1983) (var. *Monochoriavaginalisplantaginea* (Roxb.) Solms);Patwary et al. (1989)				
Commelinales	Pontederiaceae	*Monochoria vaginalis* (Burm.f.) C. Presl ex Kunth	2n = 48			Wang and Kusanagi (1996) (var. *Monochoriavaginalisangustifolia* G.X.Wang)		
Commelinales	Pontederiaceae	*Monochoria vaginalis* (Burm.f.) C. Presl ex Kunth	2n = 52	Christopher (1983), Patwary et al. (1989)			Wang and Kusanagi (1996)	
Commelinales	Typhaceae	*Typha angustifolia* L.	2n = 30					Löve and Löve (1981)
Poales	Eriocaulaceae	*Eriocaulon setaceum* L.	n/a					
